# Unmasking Cancer Risk in Heritable PAH: The Hidden Role of *BMPR2* in Epithelial Tumorigenesis

**DOI:** 10.1016/j.jaccas.2025.105344

**Published:** 2025-09-09

**Authors:** Belén Biscotti Rodil, Alejandro Cruz-Utrilla, Irene Martín de Miguel, Jorge Barriuso, Fernando Arribas Ynsaurriaga, Pilar Escribano Subías

**Affiliations:** a Pulmonary Hypertension Multidisciplinary Unit, Cardiology Department, Hospital Universitario 12 de Octubre, and CIBERCV, Madrid, Spain; b Instituto de Investigación Sanitaria Hospital 12 de Octubre (imas12), Madrid, Spain; c Oncology Department, Hospital Universitario 12 de Octubre, Madrid, Spain; d Cardiology Department, Hospital Universitario 12 de Octubre, and CIBERCV, Madrid, Spain

**Keywords:** cancer, pulmonary arterial hypertension, right ventricle

## Abstract

**Background:**

*BMPR2* mutations cause heritable pulmonary arterial hypertension (PAH) and may also influence epithelial carcinogenesis.

**Case Summary:**

We report 3 women with *BMPR2*-related PAH who developed early onset epithelial cancers: 2 breast cancers (34 and 54 years of age) and 1 colorectal cancer (47 years of age). All were on advanced PAH therapy at diagnosis. Genetic screening of relatives was negative, and additional oncogenetic testing excluded *BRCA* and Lynch syndrome. Management included chemotherapy, surgery in 2 cases, and palliative care in 1 case.

**Discussion:**

*BMPR2*, essential for vascular homeostasis, is also implicated in tumor suppression and modulation of the tumor microenvironment. In our cohort of 32 patients, epithelial cancer prevalence was 9.4%, with diagnosis at significantly younger ages than the general population. These findings suggest a potential oncogenic role for *BMPR2* mutations and the need for further research.

**Take-Home Message:**

*BMPR2* mutations may predispose to early onset epithelial cancers, warranting investigation into cancer risk and screening strategies in this population.

## Graphical Abstract

**Figure:**
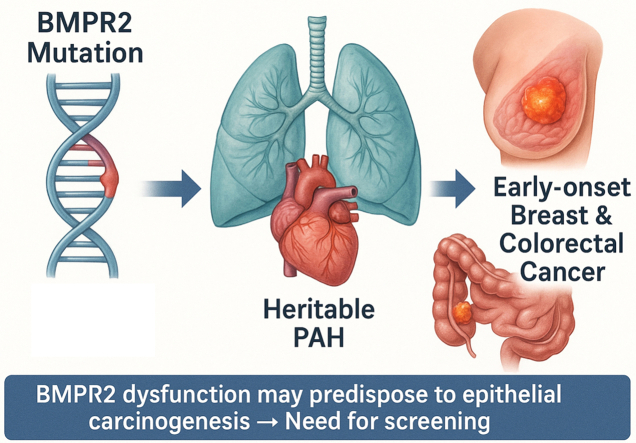

We present 3 cases of epithelial neoplasms in 3 women with heritable pulmonary arterial hypertension (PAH) associated with mutations in the *BMPR2* gene under advanced therapy, all diagnosed at an early age. The neoplasms correspond to 2 cases of breast cancer at 34 and 54 years of age, and 1 case of colorectal cancer at 47 years of age.

**Take-Home Messages**

• Mutations in the *BMPR2* gene, classically associated with heritable pulmonary arterial hypertension, may also predispose to early onset epithelial cancers.
• We report 3 cases of breast and colorectal cancer in women with *BMPR2*-related PAH, all diagnosed at younger-than-expected ages.
• These findings support the need for further investigation into cancer risk and screening strategies in this patient population.

The first case involves a 34-year-old woman diagnosed with heritable PAH due to a pathogenic *BMPR2* mutation in 2017 after an episode of hemoptysis. Dual oral therapy with tadalafil and ambrisentan was initiated at diagnosis, and she remained at low risk on this regimen until 2022, when selexipag was added. In April 2024, she experienced a new episode of hemoptysis requiring embolization. Given the echocardiographic and hemodynamic deterioration, subcutaneous treprostinil was initiated and titrated to 32 ng/kg/min. In October of the same year, she was diagnosed with triple-negative infiltrating ductal carcinoma (T3N1M0) and received chemotherapy with carboplatin, paclitaxel, and pembrolizumab. She underwent surgery without complications in March 2025.

The second case is a 54-year-old woman with no comorbidities, diagnosed with heritable PAH due to a *BMPR2* mutation in 2012. She was on triple therapy including treprostinil at a dose of 60 ng/kg/min; clinically she was at low risk but with severe hemodynamic impairment and signs of suboptimal right ventricular remodeling. Sotatercept was added to her regimen in January 2025. In March, she was diagnosed with *HER2*-negative infiltrating ductal carcinoma (T2N0M0) and started neoadjuvant chemotherapy with paclitaxel, trastuzumab, and pertuzumab. She is currently awaiting hemodynamic reassessment for surgical intervention.

The third case concerns a 47-year-old woman with heritable PAH due to a *BMPR2* mutation diagnosed in 2006 and a history of atrial flutter treated with ablation in 2023. She was on quadruple therapy (tadalafil, ambrisentan, treprostinil at 62 ng/kg/min, and sotatercept since September 2021). After constitutional symptoms and iron deficiency anemia, she was diagnosed in July 2024 with colorectal cancer with hepatic and pulmonary metastases at presentation. She began palliative treatment with raltitrexed/oxaliplatin combination, achieving partial response after 4 cycles. She was admitted in January 2025 for heart failure with refractory congestion despite aggressive diuretic treatment. She died on January 26, 2025.

As part of routine care in our center, first-degree relatives of all 3 patients underwent genetic screening, and none were found to carry the *BMPR2* mutation identified in the probands. Additionally, oncologic evaluation excluded other hereditary cancer syndromes: both patients with breast cancer tested negative for pathogenic variants in *BRCA1* and *BRCA2*, and the patient with colorectal cancer tested negative for mutations associated with Lynch syndrome and familial adenomatous polyposis.

A summary of clinical, hemodynamic, and oncologic characteristics of the 3 cases is presented in Table 1.

**Table 1:** Clinical, Hemodynamic, and Oncologic Characteristics of 3 Women With *BMPR2*-Associated Heritable Pulmonary Arterial Hypertension and Epithelial Neoplasms

	Case 1	Case 2	Case 3
Sex	Woman	Woman	Woman
Date of birth	May 1, 1990	July 16, 1970	December 20, 1977
PAH diagnosis	2017	2015	2006
PVR at PAH diagnosis, WU	11	15	27
mPAP at diagnosis, mm Hg	59	62	91
RAP at diagnosis, mm Hg	9	11	12
PCWP at diagnosis, mm Hg	8	11	18
Cancer diagnosis	October 1, 2024	March 1, 2025	August 1, 2024
Age at cancer diagnosis, y	34	54	47
Type of cancer	Breast	Breast	Metastatic colon
Cancer treatment	Chemotherapy + surgery	Chemotherapy, surgery pending	Palliative chemotherapy
PAH treatment	Tadalafil 40 mg + ambrisentan 10 mg + treprostinil 32 ng/kg/min (added April 2024)	Sildenafil 60 mg/8 h + macitentan + treprostinil 60 ng/kg/min + sotatercept (started January 2025)	Tadalafil 40 mg + ambrisentan 10 mg + treprostinil 62 ng/kg/min + sotatercept (started September 2021)
Last RHC	February 13, 2025	January 2024	2021
PVR at last RHC, WU	5	11	6.7
mPAP at last RHC, mm Hg	50	61	38
RAP at last RHC, mm Hg	7	6	4
WHO FC at cancer diagnosis	I	II	III
6MWD at cancer diagnosis, m	504	519	492
proBNP at cancer diagnosis, pg/mL	46	71	292
COMPERA 2.0: 4-risk strata	1	1	2
TAPSE at cancer diagnosis, mm	20	25	22.5
FAC at cancer diagnosis, %	34	36	38
sPAP at cancer diagnosis, mm Hg	Not measurable	46	56
TAPSE/sPAP at cancer diagnosis	Not measurable	0.54	0.40

6MWD = 6-minute walk distance; FAC = fractional area change; mPAP = mean pulmonary arterial pressure; PAH = pulmonary arterial hypertension; PCWP = pulmonary capillary wedge pressure; proBNP = pro–brain natriuretic peptide; PVR = pulmonary vascular resistance; RAP = right atrial pressure; RHC = right heart catheterization; sPAP = systolic pulmonary arterial pressure; TAPSE = tricuspid annular plane systolic excursion; WHO FC = World Health Organization Functional Class.

## Discussion

PAH is a progressive and potentially fatal vascular disease characterized by increased pulmonary vascular resistance leading to right ventricular dysfunction.^1^ In up to 80% of familial cases and 15% to 40% of sporadic cases, mutations in the *BMPR2* gene are identified. *BMPR2* encodes a receptor of the transforming growth factor beta signaling pathway, which is essential for vascular homeostasis. Its dysfunction is associated with pathologic vascular remodeling and endothelial dysfunction.^2^

Although the role of *BMPR2* in vascular pathophysiology is well established, recent studies suggest a potential involvement in neoplastic processes, particularly of epithelial tissues such as the colon and breast. In these tissues, *BMPR2* participates in the regulation of cell proliferation, differentiation, and local immune responses. This intersection between vascular signaling and carcinogenesis raises new hypotheses regarding cancer risk in *BMPR2* mutation carriers.

In colorectal cancer, *BMPR2* acts as a tumor suppressor gene. Its loss or inactivation has been linked to hereditary predisposition to polyp development and nonclassical polyposis syndromes unrelated to common mutations in genes such as *APC* or *MLH1*.^3^ Furthermore, *BMPR2* inactivation has been described in advanced stages of the adenoma-carcinoma sequence, and certain BMPR2 polymorphisms have been associated with increased colorectal cancer risk.^4^ Additionally, loss of *BMP2*-mediated signaling in the intestinal epithelium promotes a microenvironment conducive to uncontrolled proliferation and malignant transformation.^5^

In breast cancer, *BMPR2* also exerts a suppressive function, primarily via its action in the tumor stroma. In murine models, fibroblast-specific deletion of *BMPR2* significantly increases pulmonary metastasis, associated with higher levels of proinflammatory cytokines and infiltration by myeloid cells that promote a prometastatic tumor microenvironment.^6^ This phenomenon does not correlate with increased primary tumor growth, but rather with enhanced dissemination and immune evasion, suggesting *BMPR2* acts as a negative regulator of stromal inflammation, limiting tumor progression. Conversely, in vitro functional studies have demonstrated that *BMPR2* may exert pro-oncogenic effects in certain cellular contexts, enhancing tumor cell proliferation and migration.^7^ This apparent functional duality may depend on cell type and tumor microenvironment, highlighting the complexity of its role in carcinogenesis. In this regard, *BMPR2* overexpression has also been reported in the peripheral blood of patients with advanced breast cancer, possibly reflecting altered systemic signaling or a compensatory mechanism with potential diagnostic value.^8^ Additionally, recent bioinformatic analyses have identified genetic and epigenetic alterations in *BMPR2*, *BMPR1A*, and *BMPR1B*, including somatic mutations and aberrant methylation, associated with lower gene expression and worse prognosis in metastatic breast cancer.^9^

Collectively, these findings underscore the relevance of *BMPR2* not only in vascular regulation but also as a modulator of the tumor microenvironment and cancer progression. Its loss or dysfunction may facilitate tumor initiation in the colon and promote inflammation and metastatic dissemination in the breast, thereby positioning this receptor as a critical molecular node and a potential therapeutic target or biomarker in epithelial cancers.

As a national referral center for the diagnosis, treatment, and follow-up of patients with pulmonary hypertension, our unit has identified a 9.4% prevalence of epithelial neoplasms in a cohort of 32 patients with heritable PAH due to *BMPR2* mutation. The mean age at cancer diagnosis in these cases was significantly lower than that reported in the general population. In Spain, breast cancer is the most frequent malignancy in women, accounting for 28.9% of cases with an estimated prevalence of 12.5% among females, whereas colorectal cancer is the most prevalent malignancy when considering both sexes, corresponding to 15% of all cancer diagnoses and a general population prevalence of 0.9%.^10^ The average age at diagnosis is 56 years for breast cancer and 70 years for colorectal cancer.^10^ In contrast, our patients developed these neoplasms at markedly younger ages (34, 47, and 54 years), supporting the hypothesis of a potential cancer predisposition in patients with alterations in the *BMPR2* pathway.

**Figure 1:**
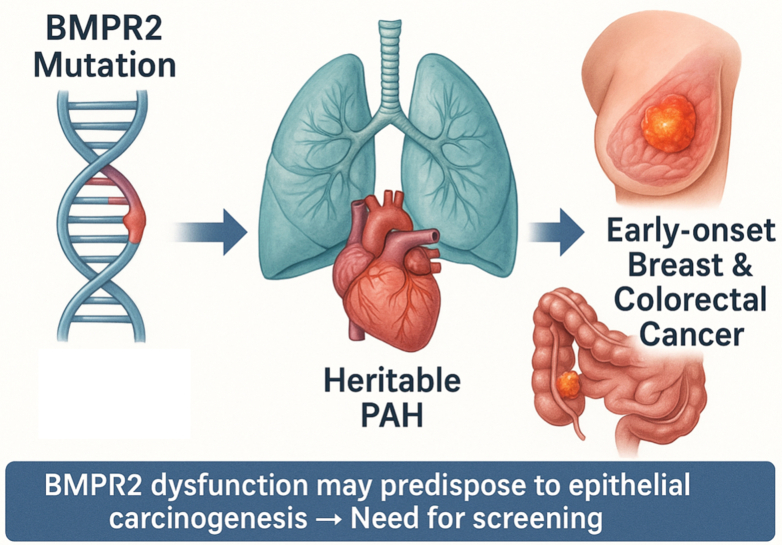
Proposed Link Between *BMPR2* Mutations, PAH, and Early Onset Epithelial Cancer PAH = pulmonary arterial hypertension.

Although breast and colorectal cancers are among the most common malignancies in the general population, the unusually early age at diagnosis observed in our patients, together with growing evidence on the role of *BMPR2* in regulating cell proliferation, inflammatory signaling, and modulation of the tumor microenvironment, suggests a potential association between *BMPR2* mutations and the development of epithelial tumors. Our clinical findings, consistent with existing biological data, support the hypothesis that *BMPR2* dysfunction may predispose carriers to early onset epithelial carcinogenesis. Whether these patients require early screening and targeted oncologic surveillance strategies remains to be elucidated. In any case, translational research focused on the oncogenic role of *BMPR2* should be promoted. Further research and international multicenter collaborations among expert pulmonary hypertension units are warranted to expand the number of reported cases, consolidate evidence, and enable statistically robust analyses to clarify this emerging and potentially clinically relevant association (Figure 1).

## Conclusions

Our findings suggest that *BMPR2* mutations may predispose to early onset epithelial cancers in patients with heritable PAH. Further research is needed to define cancer risk and establish appropriate surveillance strategies.

## Funding Support and Author Disclosures

Dr Martín de Miguel is supported by a Río Hortega Grant from the Instituto de Salud Carlos III (ISCIII, CM23/00235). Dr Cruz-Utrilla is supported by a Juan Rodés Grant from the Instituto de Salud Carlos III (ISCIII, JR23/00071). All other authors have reported that they have no relationships relevant to the contents of this paper to disclose.
